# In silico prediction and expression profile analysis of small non-coding RNAs in *Herbaspirillum seropedicae* SmR1

**DOI:** 10.1186/s12864-019-6402-x

**Published:** 2020-02-10

**Authors:** Tatiane Dobrzanski, Vânia Pobre, Leandro Ferreira Moreno, Helba Cirino de Souza Barbosa, Rose Adele Monteiro, Fábio de Oliveira Pedrosa, Emanuel Maltempi de Souza, Cecília Maria Arraiano, Maria Berenice Reynaud Steffens

**Affiliations:** 1 Department of Biochemistry and Molecular Biology, Universidade Federal do Paraná (UFPR), Av. Coronel. Francisco H. dos Santos, 210, PoBox 19046, Curitiba, 81.531-980 Paraná Brazil; 2 Instituto de Tecnologia Química e Biológica António Xavier, Universidade Nova de Lisboa, Av. da República, 2780-157 Oeiras, Portugal; 3 Graduate Program in Bioinformatics, Universidade Federal do Paraná (UFPR), Rua Alcides Vieira Arcoverde, 1225, Curitiba, 81520-260 Brazil

**Keywords:** *Herbaspirillum seropedicae* SmR1, sRNA, Regulatory RNA, Nitrogen fixation, Diazotrophic bacterium, Bacterial plant interaction

## Abstract

**Background:**

*Herbaspirillum seropedicae* is a diazotrophic bacterium from the β-proteobacteria class that colonizes endophytically important gramineous species, promotes their growth through phytohormone-dependent stimulation and can express *nif* genes and fix nitrogen inside plant tissues. Due to these properties this bacterium has great potential as a commercial inoculant for agriculture. The *H. seropedicae* SmR1 genome is completely sequenced and annotated but despite the availability of diverse structural and functional analysis of this genome, studies involving small non-coding RNAs (sRNAs) has not yet been done. We have conducted computational prediction and RNA-seq analysis to select and confirm the expression of sRNA genes in the *H. seropedicae* SmR1 genome, in the presence of two nitrogen independent sources and in presence of naringenin, a flavonoid secreted by some plants.

**Results:**

This approach resulted in a set of 117 sRNAs distributed in riboswitch, *cis*-encoded and *trans*-encoded categories and among them 20 have Rfam homologs. The housekeeping sRNAs tmRNA, ssrS and 4.5S were found and we observed that a large number of sRNAs are more expressed in the nitrate condition rather than the control condition and in the presence of naringenin. Some sRNAs expression were confirmed in vitro and this work contributes to better understand the post transcriptional regulation in this bacterium.

**Conclusions:**

*H. seropedicae* SmR1 express sRNAs in the presence of two nitrogen sources and/or in the presence of naringenin. The functions of most of these sRNAs remains unknown but their existence in this bacterium confirms the evidence that sRNAs are involved in many different cellular activities to adapt to nutritional and environmental changes.

## Background

*Herbaspirillum seropedicae* SmR1 is a diazotrophic and endophytic bacterium that belongs to the β-proteobacteria. This microorganism fixes nitrogen under microaerobic conditions inside the plant tissues of the economically important cereal crops wheat, rice, maize and sorghum [1]. *H. seropedicae* strains also appears associated with sugar cane and forage grasses [2, 3], fruit crops [4] and common bean [5]. Several studies have demonstrated the benefits of *Herbaspirillum*-plant interaction through the increase of the biomass of the inoculated plant [6–10]. Nitrogen cellular sources are the atmospheric dinitrogen and the nitrate, present in several environments. *H. seropedicae* SmR1 fixes nitrogen to ammonia in a reaction catalysed by the enzyme nitrogenase [11, 12]. The bacterial-plant interaction can promote plant growth and increase yield of crops since some compounds produced by the bacterium can stimulate the synthesis of phytohormones by plants [13]. Plants also play an important role in establishing this interaction since they produce compounds that affect their associations with microorganisms. One of such compounds is naringenin, a flavonoid produced as secondary metabolite, that can stimulate or inhibit specific genetic responses in different bacteria associated with plants [14, 15]. It was demonstrated that naringenin stimulates the endophytic colonization of *Arabidopsis thaliana* by *H. seropedicae* Z67 [16]. In *H. seropedicae* SmR1, naringenin regulates the expression of several genes, positively or negatively [17]. This microorganism can catabolize naringenin probably to obtain carbon and energy [18].

The single circular chromosome of the *H. seropedicae* SmR1 strain was sequenced and 4804 open reading frames were annotated [19]. Since then, there are many studies focusing the genomic structure, gene expression and physiology of *H. seropedicae* SmR1 [12, 17, 18, 20–22], but the investigation about the presence and function of small non-coding RNAs (sRNAs) was never performed. sRNAs have key regulatory roles in post-transcriptional control of gene expression. They can modulate turnover of target mRNAs and affect their translation [23–25]. They can be found in all three domains of life and are particularly important in bacteria allowing them to rapidly respond to environmental challenges [26, 27]. These molecules are 50–500 nucleotides long and are located predominantly in intergenic or in untranslated regions in the bacterial genomes [28]. sRNAs can be divided in two major categories *trans*-encoded RNAs (traRNA) and *cis*-encoded RNAs (caRNA) [29]. The caRNA can act at transcriptional or translational level and are sensory RNAs elements such as riboswitches that adopt two conformational structures in response to chemical signals such as small ligands [30–33]. The traRNA comprises the *trans*-encoded sRNAs that are partially complementary to their target [23] and the antisense small RNAs (asRNAs) that are totally complementary to their target [34, 35]. There are still traRNAs that bind to proteins to modulate their activity such as 6S RNA [36]. The paring of many traRNAS to mRNA target sites is facilitated by the RNA chaperone Hfq, a Sm family protein, which binds to adenine- and uridine-rich sequences (AU-motif) in sRNA [37, 38]. *H. seropedicae* SmR1 contains a conserved Hfq protein with a classic hexameric ring shape, observed in all available Hfq structures, with sRNA and mRNA contact surfaces [39]. The presence of a variety of types of small non-coding RNAs provides a versatile regulation of metabolic functions [25, 40–42]. The bioinformatic prediction of sRNAs followed by RNA-seq approach made possible genome screens for sRNAs and has shown that there are much more bacterial regulatory sRNAs than previously thought [43]. In this study we applied in silico approach to predict sRNAs in *H. seropedicae* SmR1 genome and RNA-seq analysis to confirm their expression in bacteria grown in the presence of two nitrogen sources (ammonia or nitrate) and in the presence of naringenin. A set of 117 sRNAs transcripts were confirmed and some of them showed sequence identity with well-characterized sRNAs in other bacteria. Some sRNAs were experimentally detected confirming their existence.

## Results

### sRNAs in the *H. seropedicae* SmR1 genome

**Fig. 1:**
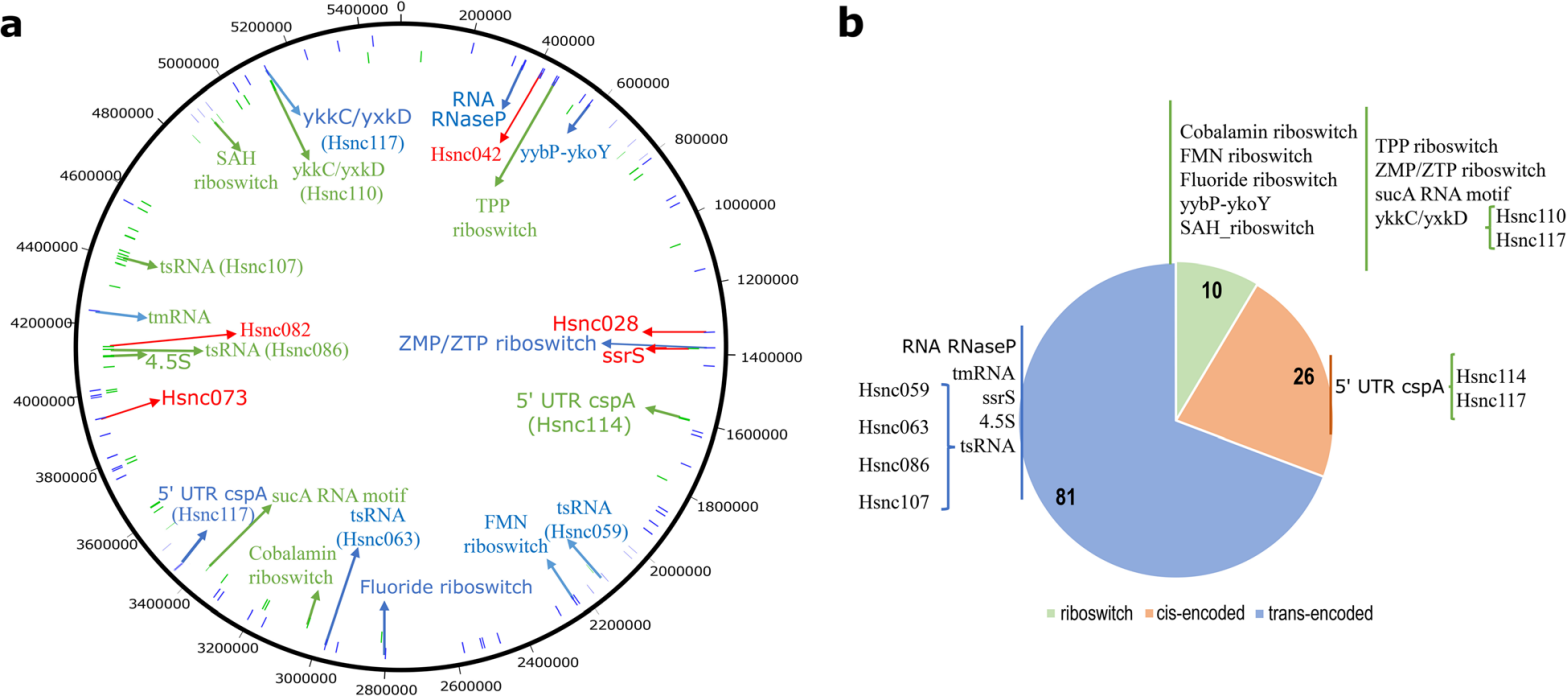
Identification of the sRNAs predicted by bioinformatics of *H. seropedicae SmR1*. **a** Distribution of 117 sRNAs in the genome of *H. seropedicae* SmR1. The sRNAs annotated on the sense DNA strand are marked in blue and the annotated on the antisense strand in green. The sRNAs with defined identity (homology given by Rfam) are indicated in green or blue and the selected for experimental validation are indicated in red.** b** Categorization of predicted sRNA of *H. seropedicae* SmR1. 26 cisencoded, 81 trans-encoded and 10 riboswitch sRNAs were identified and those with RFAM identity in each group are indicated

To search sRNAs in the genome of *H. seropedicae* SmR1 we used the nocoRNAc software [44], a bioinformatic tool that predicts sRNAs based on the co-localization of transcriptional terminators and promoter and is not limited to intergenic regions [44]. We identified 769 putative sRNAs. At the same time, we verified the presence of sRNAs transcripts in the RNA-seq data of *H. seropedicae* SmR1 using Cufflinks [45]. We were able to identify 1395 regions being transcribed which could encode sRNAs. Were analysed three RNA-seq data conditions obtained during the exponential growth phase of the bacterium: (i) control (CRT) - bacteria grown in NFbHPN medium containing NH_4_Cl as nitrogen source, (ii) presence of naringenin (NAR) - bacteria cultured in NFbHPN medium containing NH_4_Cl and the flavonoid naringenin, and (iii) nitrate (NIT) - bacteria grown in NFbHP medium containing KNO_3_ as nitrogen source. Using the coverage criterion which established a minimum coverage ≥5 as a confidence level to select sRNAs in at least one of the three culture conditions we were able to verify the expression of 117 sRNAs transcripts in *H. seropedicae* SmR1 which have been termed Hsnc001 to Hsnc117 (Additional file 1). Forty sRNAs (34.5%) resulted only from Cufflinks, 63 sRNAs (54.3%) resulted only from nocoRNAc and 14 (12.1%) appeared in both approaches. These sRNAs range in length from 41 to 560 nucleotides and are equally distributed in the genome of *H. seropedicae* SmR1 being 67 annotated on the sense and 50 on the antisense strand in intergenic regions (Fig. 1a). According to the genomic location the sRNAs were distributed in riboswitch (10), *cis*-encoded (26) and *trans*-encoded (81) categories (Fig. 1b). Regarding base composition of the sRNAs we observed a range of 35 to 75% GC content, with an average of 54.97% (Additional file 1), whereas the genome has about 63.4% GC [19]. We observed that the riboswitches present high GC content (61.8%) and the known housekeeping sRNAs tmRNA, ssrS and 4.5S had 52.3, 53.7 and 65.7% GC content, respectively.

### sRNAs of *H. seropedicae* SmR1

Comparison of *H. seropedicae* SmR1 117 sRNAs transcripts with RNA family’s data base (Rfam) returned only 20 sequences with hits and information about putative function (Table 1). This result suggests that most of the *H. seropedicae* SmR1 sRNAs may be new or present low level of identity with those deposited in the Rfam database. Among the sRNAs identified the Toxic small RNA (tsRNA) and sucA RNA motif were found essentially in β-proteobacteria [46, 47] whereas YkkC/YxkD leader is present in some Cyanobacteria and Proteobacteria [48]. The sRNAs belonging to the family of small toxic RNAs in *H. seropedicae* SmR1 were Hsnc59, Hsnc63, Hsnc86 and Hsnc107 (Table 1). These small Toxic RNAs were found to be expressed in several strains of *Burkholderia cenocepacia* and, although they do not present a known function, they are capable of inhibiting *Escherichia coli* growth when introduced in a cloning vector [47, 49]. The Hsnc006 was annotated as the sucA 5’UTR which is considered a riboswitch candidate since the ligand that changes its conformation is still unknow [50]. This sequence is the 5′ UTR of *sucAsucBlpd* operon and, according to RNA-seq data, the RNA motif and the operon exhibited proportional expression level in the three conditions analysed (Additional file 1). We found two copies of YkkC/YxkD leader (Hsnc110 and Hsnc116) upstream HSERO_RS22365 and HSERO_RS22370, respectively, which encode two lipid kinases involved in the inorganic ion transport and metabolism. Recently this riboswitch was renamed as guanidin-I riboswitch since it senses and responds to guanidine and controls genes that modify or pump guanidine as a toxic compound of bacteria [51].

**Table 1:** *H. seropedicae* SmR1 sRNAs identified in Rfam

Predicted			Rfam					
sRNA	size	GC%	ID	Acession	Start	End	Bit score	e-value
Hsnc001	99	65.66	4.5S	RF00169	1	99	76.4	8E-19
Hsnc002	111	60.36	Fluoride riboswitch	RF01734	8	81	50.3	4.4e-10
Hsnc006	94	56.38	sucA RNA motif	RF01070	12	93	79.8	2.3e-16
Hsnc029	100	61.00	ZMP/ZTP riboswitch	RF01750	1	100	59.3	2.6e-09
Hsnc035	169	65.09	FMN riboswitch	RF00050	1	169	112.6	5.3e-28
Hsnc050	177	53.67	ssrS (6S)	RF00013	1	177	67.3	2.5e-14
Hsnc059	93	49.46	Betaproteobacteria toxic RNA	RF02278	25	93	59.6	1.4e-12
Hsnc063	115	43.48	Betaproteobacteria toxic RNA	RF02278	48	115	62.4	3.4e-13
Hsnc083	384	52.34	tmRNA	RF00023	1	381	193.0	4.6e-57
Hsnc086	97	47.42	Betaproteobacteria toxic sRNA	RF02278	34	97	64.7	7.6e-14
Hsnc107	98	43.88	Betaproteobacteria toxic sRNA	RF02278	31	97	61.1	6.1e-13
Hsnc109	293	58.36	TPP riboswitch	RF00059	86	196	55.6	2.3e-10
Hsnc110	101	64.36	ykkC-yxkD	RF00442	1	101	99.0	1.8e-23
Hsnc111	335	61.19	RNA RNaseP	RF00010	1	335	212.1	1.1e-68
Hsnc112	184	63.04	yybP-ykoY	RF00080	16	184	47.7	6E-12
Hsnc113	90	65.56	SAH riboswitch	RF01057	1	90	49.1	9.6e-09
Hsnc114	373	51.47	5′ UTR cspA	RF01766	1	373	94.3	1.6e-24
Hsnc115	247	63.97	Cobalamin riboswitch	RF00174	1	247	111.2	1.6e-30
Hsnc116	100	60.0	ykkC-yxkD	RF00442	1	100	93.4	4.8e-22
Hsnc117	387	48.97	5′ UTR cspA	RF01766	1	388	86.7	2.7e-22

**Table 2:** Oligonucleotides used in radiolabelling reactions

Probe	Sequence (5′ – 3′)
ssrS-F	CCGTGTTCGCGATTGCC
ssrS-T7	TAATACGACTCACTATAGGCCGGCATCCTGAACCTG
Hsnc042-F	GATGCCCGACTGCTGAAACG
Hsnc042-T7	TAATACGACTCACTATAGGTAGCGTCGGAATCGCGTTCCTG
Hsnc073-F	GCAATAACCAATGCGCAGG
Hsnc073-T7	TAATACGACTCACTATAGGGCATCATCAAGGGATGCCAG
Hsnc028	AAATCAGGCGTTTGTCATGGTTCGGTAAG
Hsnc082	AACGATGGAAGTACGGTGGTTCGCGTGATG

The T7 promoter sequence in the oligos is underlined

We also found the TPP (Hsnc109) and FMN (Hsnc035) riboswitches. The TPP riboswitch is immediately upstream of HSERO_RS02120 (*thiC*) encoding the phosphomethylpyrimidine synthase and is known to bind directly to thiamine pyrophosphate (TPP) turning off TPP biosynthesis [52]. The FMN riboswitch is upstream of HSERO_RS09820 (*ribE*) encoding the 6,7-dimethyl-8-ribitylllumazine synthase which catalyses one of last steps in the biosynthesis of riboflavin. FMN binds to the FMN aptamer and regulates the *ribE* expression [53]. *H. seropedicae* SmR1 still presents the SAH riboswitch (Hsnc113), Cobalamin riboswitch (Hsnc115), ZMP / ZTP riboswitch (Hsnc029), Yybp-ykoY (Hsnc112) and Fluoride riboswitch (Hsnc002). The SAH riboswitch is upstream HSERO_RS21435, encoding S-adenosyl- (L) -homocysteine (SAH), and is involved in S-adenosyl- (L) -methionine (SAM) regeneration cycle [54, 55] Cobalamin riboswitch is upstream HSERO_RS13325-HSERO_RS13320 operon encoding the cobalt transporter CbtB-CbtA acting in concert with vitamin B12 biosynthesis systems [56, 57]. ZMP/ZTP riboswitch regulates the expression of carbon metabolism genes [58, 59]. *H. seropedicae* SmR1 showed this riboswitch (Hsnc029) upstream to the HSERO_RS06140 (*glyA*) encoding serine hydroxymethyltransferase, a pyridoxal phosphate-dependent enzyme that plays an important role in the cellular pathways of a carbon [59].

Yybp-ykoY is a manganese riboswitch that binds directly to Mn^2+^ and is associated with YebN/MntP genes [60–62]. In *H. seropedicae* SmR1, this riboswitch is located upstream HSERO_RS02630 encoding the manganese efflux pump MntP. In *Xanthomonas oryzae* this riboswitch acts as an essential Mn^2+^ sensor in infections during interaction with rice [61]. Fluoride riboswitch located upstream of HSERO_RS12335 coding the voltage-gated chloride channel protein. This riboswitch has been experimentally verified by [63] detecting fluoride and triggering the expression of genes that can help *Enterobacter cloacae* FRM to mitigate fluoride toxicity, using a fluorine carrier to expel fluoride from the cells.

We found only two sRNAs with hits in the *cis*-encoded category, the *cspA* 5’UTR mRNAs Hsnc114 and Hsnc117. These sRNAs were identified in *H. seropedicae* SmR1 in untranslated regions of genes HSERO_RS07020 and HSERO_RS15195 encoding cold-shock proteins. These elements are known to be involved in the expression of *cspA* in response to temperature shift [64, 65] however it was already demonstrated that they might have a role in stress tolerance [66]. Both *H. seropedicae* cspA 5 ‘UTR mRNAs contain single-strand AU-motif in mRNA which could be binding sites of the RNA chaperone Hfq, as demonstrated in *E. coli* [67].

Some well-preserved housekeeping sRNAs were also found in *H. seropedicae* SmR1, ssrS or 6S RNA (Hsnc050), 4.5S RNA (Hsnc001) and tmRNA (Hsnc083). These sRNAs associate with proteins and are highly expressed in the cell. The 6S interacts with the primary form of holoenzyme of RNA polymerase, negatively regulates transcription and is involved in modulating stress and optimizing survival during nutrient limitation [68]. The 4.5S is part of the signal recognition particle (SRP) ribonucleoprotein complex [69]. In most bacteria the SRP consists of an RNA molecule (4.5S) and the Ffh protein that bind to ribosome stopping protein synthesis. The tmRNA (transfer messenger RNA) forms a ribonucleoprotein complex (tmRNP) that binds to bacterial ribosomes that are blocked in the middle of protein synthesis; it is able to recycle the blocked ribosome by bringing a stop codon and g a proteolysis-inducing tag to unfinished polypeptides [70].

### Expression of sRNA in *H. seropedicae* SmR1

**Fig. 2:**
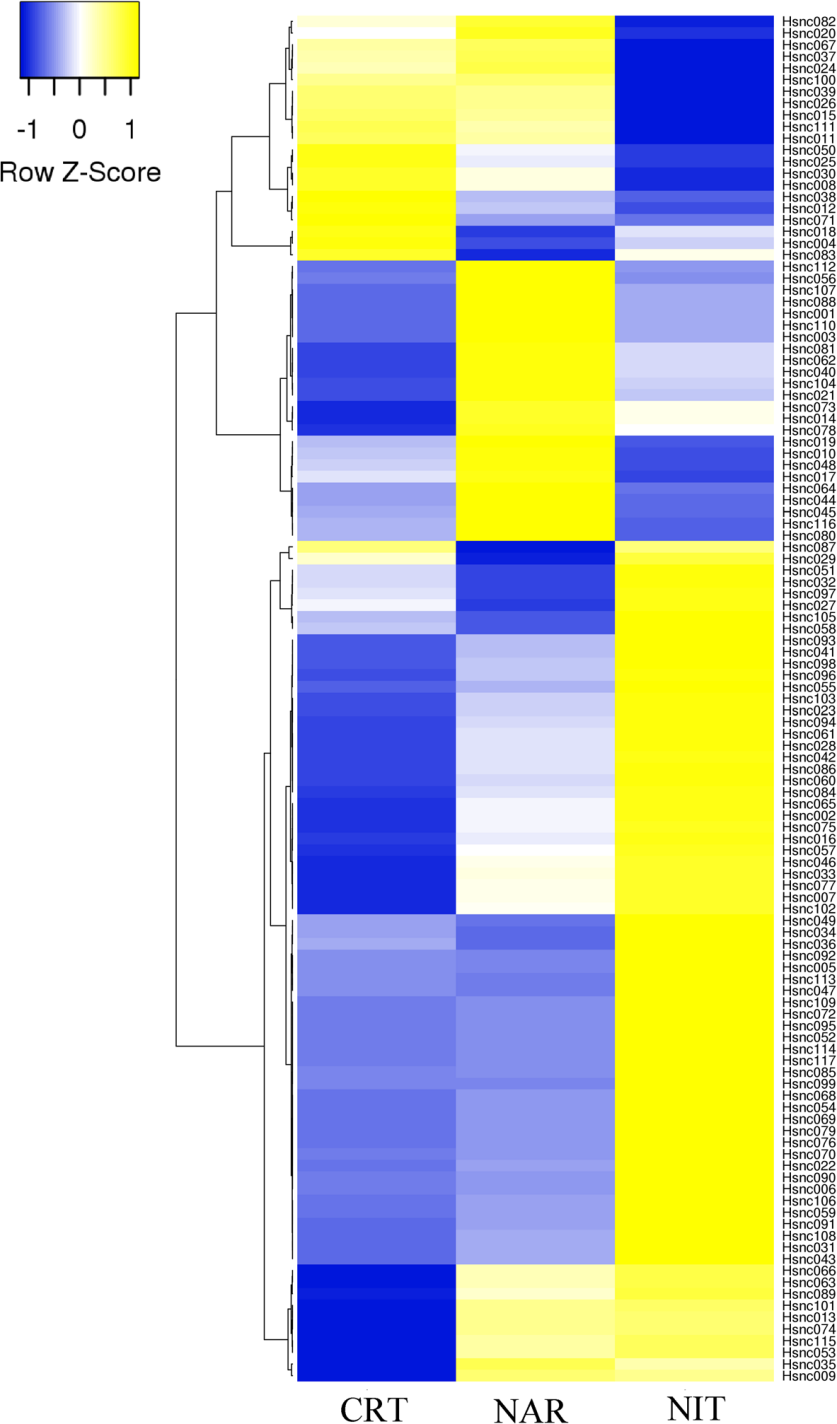
Heatmap showing relative expression levels of 117 sRNAs in the genome of *H. seropedicae* SmR1. The heatmap showed the expression levels of sRNAs in the control culture condition (CRT), presence of naringenin (NAR) and nitrate (NIT) during the exponential phase. The dendrogram provides the visualization of a hierarchical clustering of sRNAs with similar expression patterns. A scale of z-score relation to colour intensity is shown

Bacteria have a versatile system to respond quickly to environmental changes. In this process, many sRNAs are often expressed to regulate gene expression in a specific or different conditions and stages of growth. Thus, we wanted to determine how sRNAs are expressed in different culture conditions. The reading count of exponential phase in RNA-seq data was performed for each sRNA followed by normalization by Reads Per Kilobase Million (RPKM). A heat map that includes the 117 sRNA reveals different expression profiles according to the growth condition of *H. seropedicae* SmR1 (Fig. 2). We observed that a large cluster with 62 sRNAs are more expressed in the nitrate condition than in the control and naringenin conditions. Another cluster with 24 sRNAs is more expressed in the naringenin condition than in the control and nitrate conditions. This demonstrates the role of environment in the expression of sRNAs and the influence of sRNAs when the bacterium is exposed to certain nutritional conditions.

### Experimental validation of *H. seropedicae* SmR1 sRNAs

We also used the coverage criterion of RNA-seq reads greater than or equal to five, in at least one of the three culture conditions (Additional file 1), to select Hsnc050, Hsnc028, Hsnc042, Hsnc073 and Hsnc082 sRNAs and validate their expression by Northern blot. Although coverage values do not represent expression quantification (as opposed to RPKM quantification), they can indicate that a given sRNA may be expressed. ssrS (Hsnc050) was chose as a control since it is a conserved housekeeping sRNA among bacterial species [68, 71, 72].

**Fig. 3:**
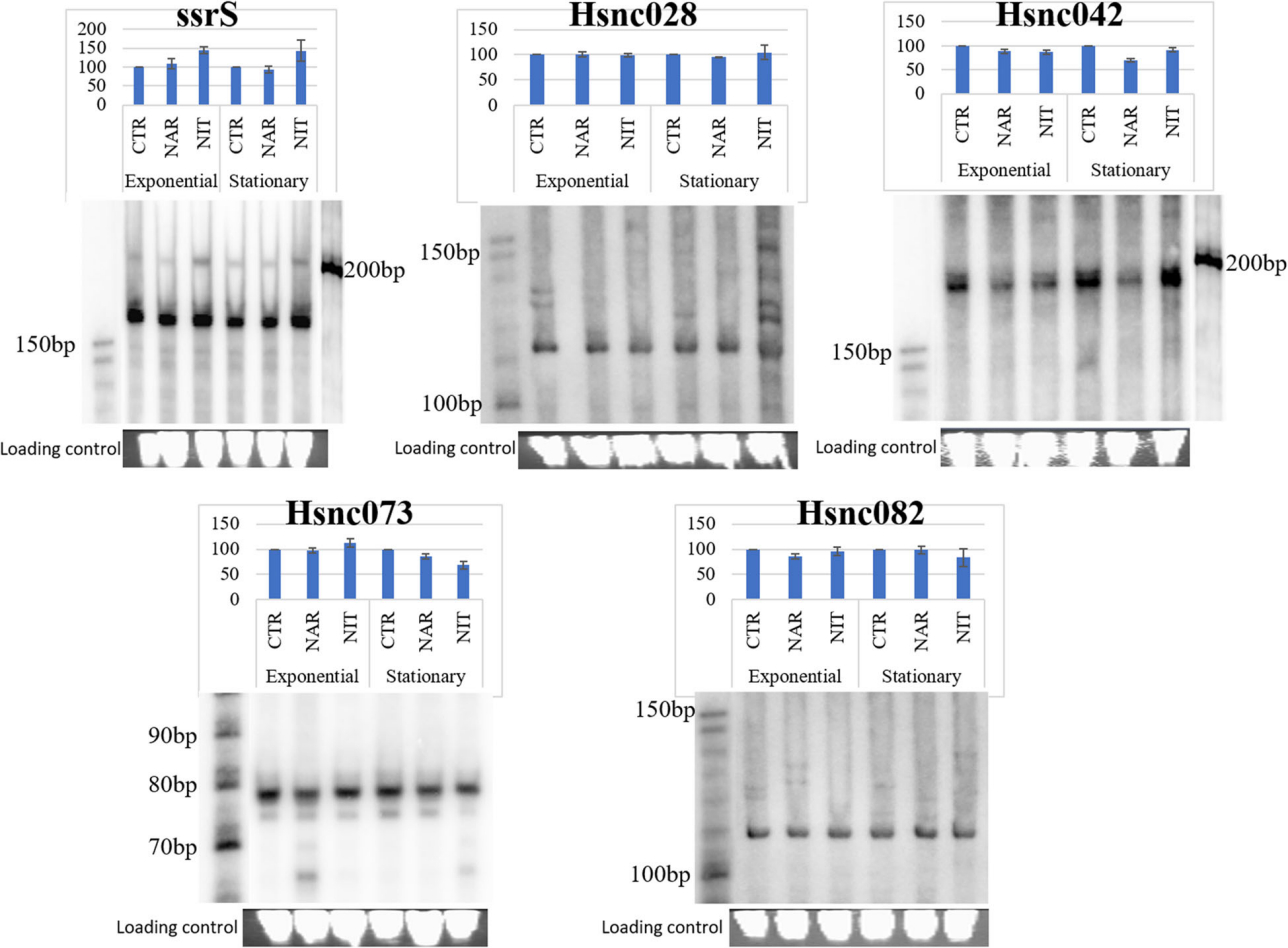
Validation of ssrS, Hsnc028, Hsnc042, Hsnc073 and Hsnc082 sRNAs of *H. seropedicae* SmR1 by northern blot. The expression of sRNAs was analysed in the exponential phase (OD_600_ ~ 0.7) and in the stationary phase (after 10 h growth), in the control condition (CRT), in the presence of naringenin (NAR) and in the nitrate (NIT) condition. Markers are indicated to the right and left in the images. Quantification of northern blot gels is above each northern blot image. The CRT condition was defined as the standard for the quantifications, therefore, it presents a value of 100% for each growth phase, whereas the NAR and NIT conditions vary in relation to the CRT

Considering that many sRNAs are induced under stress conditions, such as the lack of nutrients in the stationary phase, we evaluate the expression of the sRNAs in two phases of growth, exponential (OD_600_ = 0.7) and stationary phase (after 10 h of culture). *H. seropedicae* SmR1 was cultured under the CRT, NAR and NIT conditions and the total RNA was extracted and hybridized with specific radiolabelled probes. We were able to confirm that the five selected sRNAs are expressed in *H. seropedicae* SmR1 (Fig. 3). It is notable that all RNAs were expressed in all analysed growth conditions (CRT, NAR and NIT) as well as in the exponential and stationary phases (Fig. 3). We quantified at least three replicates of northern blots for each sRNA that was experimentally validated. The CRT conditions of each growth phase were used as the standard of comparison between the different treatments. The expression of sRNAs did not show significant differences between culture conditions and growth phases (Fig. 3). However, a small decrease in expression for the Hsnc042 sRNA in the NAR condition in stationary phase and for Hsnc073 sRNA under NAR and NIT conditions in stationary phase was observed.

**Fig. 4:**
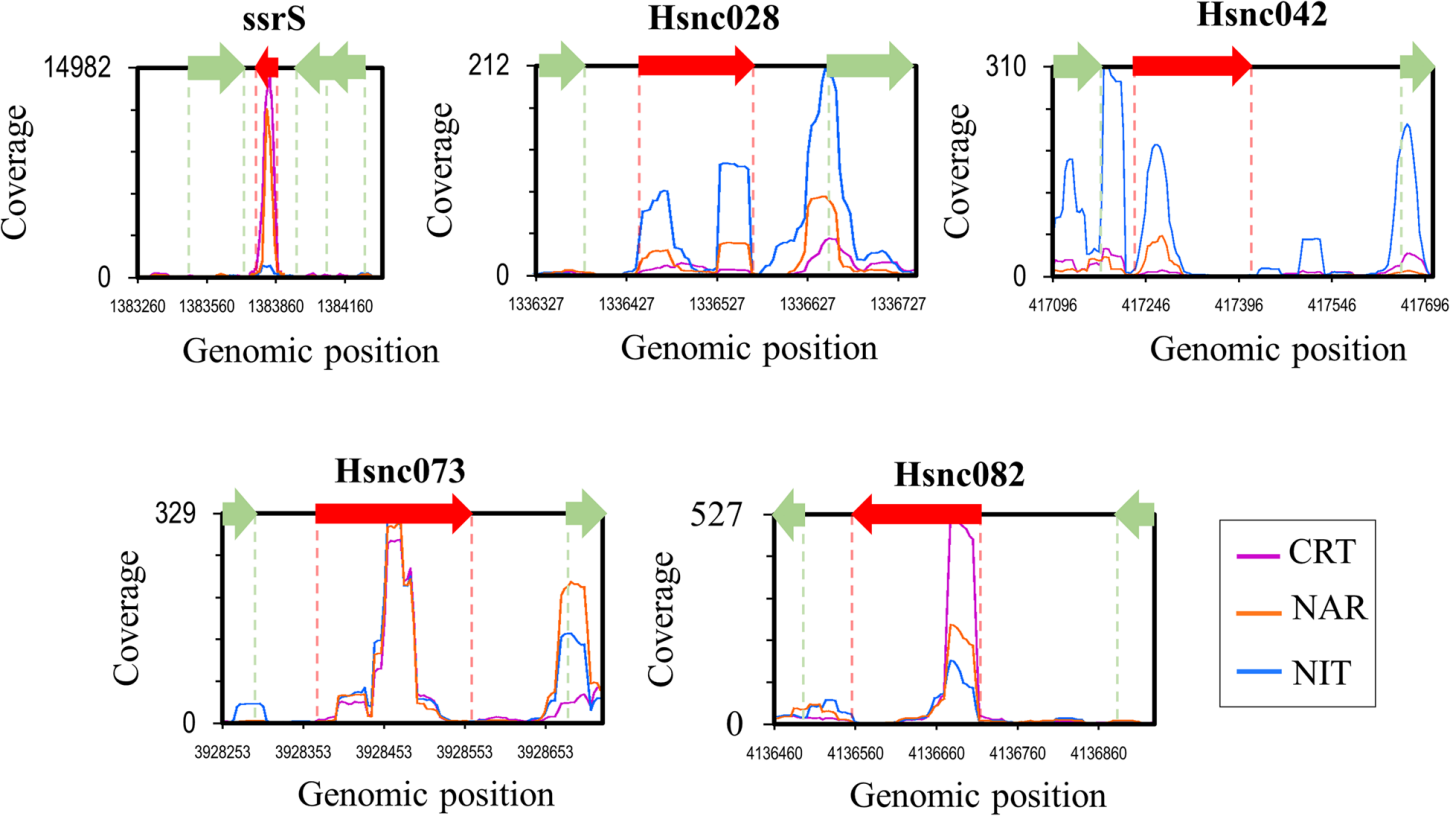
Coverage profiles of *H. seropedicae* SmR1 sRNAs in the exponential phase detected by RNA-seq. The y-axes indicate the reading coverage for each sRNA in the control conditions (purple lines), in the presence of naringenin (orange lines) and nitrate (blue lines). The x-axes denote the genomic positions according to the *H. seropedicae* SmR1 genome coordinates

The function of these sRNAs is yet to be elucidated. In addition, we notice that Hsnc073 and Hsnc082 sRNAs showed lengths in the northern blot smaller (~ 78 nt and ~ 120 nt respectively) than the length initially predicted by bioinformatics (182 nt and 157 nt respectively) (Fig 4). We suggest the smaller lengths of Hsnc073 and Hsnc082 sRNAs may be due to sRNA processing since most of the sRNAs are transcribed with larger length and then later processed by RNases for smaller functional lengths [28]. This difference can be also due to an imprecision in the prediction of sRNAs by the bioinformatics tools used in this work. Previously, we already observed that the nocoRNAc tool can predict sRNAs with larger than expected lengths, as the case of ssrS (6S). This sRNA was initially predicted with a length of 327 nucleotides by nocoRNAc, however based on RNA-seq we corrected its length to 177 nucleotides and, in fact, an RNA band with around this length was obtained in the northern blot (Fig. 3). Bacterial ssrS (6S) RNAs are generally transcribed as pre-6S RNA and then processed in 5′ end by ribonucleases that cuts a short sequence to mature form [73]. The *H. seropedicae* 6S RNA length is very close to the 6S of *Neisseria meningitidis* MC58 and *Pseudomonas aeruginosa* which are about 180 nucleotides [71]. The Hsnc028 (126 nucleotides) and Hsnc073 (182 nucleotides) also presented well distributed coverage in the RNA-seq profile (data not shown).

## Discussion

In this work we identified and validated sRNAs in β-proteobacteria *H. seropedicae* SmR1, a highly versatile diazotrophic bacterium capable of metabolizing a wide range of carbon and nitrogen sources. Initially, we showed that 2164 sRNAs were predicted by bioinformatics tools Cufflinks and nocoRNAc that uses a specific prediction method for prokaryotic sRNA. To confirm which of these sRNAs are expressed in *H. seropedicae* SmR1, we analysed RNA-seq data from three culture conditions (CRT, NAR and NIT) and verified the expression of 117 sRNAs, each expressed in at least one of the three conditions analysed. The number of sRNAs predicted by bioinformatics tools varies among species as already described for *Streptomyces coelicolor* (843 sRNAs) [44], *Burkholderia pseudomallei* (1306 sRNAs) [74] and *B. cenocepacia* J2315 (213 sRNAs) [75]. We can attribute this variation to the different methods of total RNA purification and to the sRNA prediction and validation methods employed. Concerning to *H. seropedicae* SMR1, we should also consider the stringent criterion adopted as a confidence level for sRNA expression (minimum coverage ≥5) to avoid data noise and false-positives from the biocomputational prediction. Besides that, considering the size of the *H. seropedicae* SmR1 genome and the number of ORFs, we can expect a larger number of different sRNAs being expressed in other culture metabolic conditions. When we submitted the expressed sequences of the *H. seropedicae* SmR1 sRNAs (Additional file 1) to Rfam database the sequences we found only 20 sRNAs with hit to some RNA families (Table 1). We were able to confirm the expression of about 26 *cis*-encoded sRNAs in *H. seropedicae* SmR1 e two of them had hit to the family of *cspA* sRNAs according to Rfam. All 10 riboswitches showed good Rfam alignment scores (Table 1). Of the 81 trans-encoded sRNAs, 8 showed similarity given by Rfam. The occurrence of a large number of *H. seropedicae* SmR1 sRNAs with no hit in Rfam is not surprising since the database is populated with sRNAs from the most characterized model organisms, such as *E. coli*, *Salmonella enterica* and *Staphylococcus aureus* [76], and others that are phylogenetically distant from *H. seropedicae*. Considering the genome size of this bacterium we can expect a larger number of different sRNAs being expressed in other culture metabolic conditions. Thus, we suggest that *H. seropedicae* SmR1 may exhibit specific sRNAs with closer similarity to other organisms belonging to the same class. In β-proteobacteria the sRNAs have been described in *Burkholderia* species although they were not well functionally characterized [47, 77, 78].

Many processes could be controlled by bacterial sRNAs induced by specific metabolic or environmental signals [79] or expressed constitutively under different growth conditions, such as housekeeping sRNAs (tmRNA and 4.5S) that participate in the regulation of genes that are constitutively expressed [80]. Interestingly, all *H. seropedicae* SmR1 sRNAs had a mean GC content (54.97% GC) below the mean of the genome content (63.4% GC). However, there are some sRNAs with slightly higher GC content such as riboswitches (61.81% GC) and known housekeeping sRNAs (ssrS, tmRNA and 4.5S with an average 57.22% GC). RNAs that exhibit high GC content generally exhibit a more rigid and conserved structure wich is probably necessary for the regulation of specific target molecules, such as maintenance genes or molecules that hardly evolve. In contrast, sRNAs that have a more flexible structure with a low GC content are probably involved in regulating the expression of several different genes or molecules that frequently evolve [77, 80, 81]. We have observed that the four Toxic sRNAs have low GC content (Hsnc063 43.48%, Hsnc086 47.42%, Hsnc107 43.88% and Hsnc059 49.46%). The Toxic sRNAs currently found exclusively in β-proteobacteria are *trans*-encoded and [49] and possibly interact with several mRNAs through alignment with ribosomal binding site (RBS). Then we suggest that these sRNAs have a more flexible structure with low GC content to control the regulation of different targets.

We created a heatmap to observe the expression of sRNAs between control and different culture conditions and observed a large cluster of sRNAs more expressed in the NIT condition. The main source of nitrogen for most organisms is ammonium and, in the absence or low concentration of ammonium, bacteria need to mobilize alternative nitrogen sources to maintain growth and increase chances of survival. In the absence or restriction of ammonium, *H. seropedicae* SmR1 can assimilate nitrate [12]. The RNA-seq profile revealed that the change in nitrogen source from ammonium to nitrate caused modifications in the pattern of gene expression in *H. seropedicae* SmR1, more than 37% of the genes were differentially expressed in the nitrate condition and the carbon consumption was increased [12]. Since sRNAs may play an important role in nutritional deprivation [79], our data suggest that when the nitrate is the only nitrogen source many sRNAs may be influencing the post-transcriptional regulation of genes involved in carbon and nitrogen metabolism. When *H. seropedicae* SmR1 was cultivated in the presence of nitrate Bonato and collaborators [12] reported the increased expression of citric acid cycle genes with emphasis on *sucA* and *sucB* (7.3 and 7.7 respectively). In our work we observed the sucA RNA motif (Hsnc006) is highly expressed in the presence of nitrate (10 mmol/L) when compared to the control (20 mml/L NH_4_Cl) (Additional file 1). Since this motif typically appears at 5′ end of β-proteobacteria *sucA* genes [46, 47] this could indicate a unique adaptation of the energetic metabolism of this bacterial Class to the changes in nitrogen sources. This hypothesis should be investigated.

A small cluster of sRNAs more expressed in the presence of naringenin was also observed in the heatmap. Naringenin is a plant-derived flavonoid and may act as a signal molecule during endophytic colonization by *H. seropedicae* SmR1 [82]. In this bacterium, naringenin triggers a change in gene expression to reduce motility and flagella synthesis [17]. An extensive bacterial sensory system for adaptation and survival to the plant environment was also observed during the early stages of colonization of maize [20]. Our data suggest that many sRNAs may be involved in the post-transcriptional regulation of genes related to the adaptation and endophytic colonization of *H. seropedicae* SmR1 in plants. Further investigation is required to determine if some sRNAs may be related to flagella synthesis and bacterial motility as shown in other organisms [83, 84].

We were able to validate by Northern blot the expression of the Hsnc050 (ssrS RNA), Hsnc028, Hsnc042, Hsnc073 and Hsnc082 sRNAs. According to the Northern blot Hsnc073 and Hsnc082 sRNAs had smaller lengths than those predicted by bioinformatics. As we said earlier this difference could be due to sRNA processing or even imprecision in the prediction. Klein et al. (2202) also observed a length difference of sRNAs predicted for *Pyrococcus furiosus*, of the 11 sRNAs identified in Northern blot only one showed length corresponding to the initially predicted size [85]. *Sinorhizobium meliloti* also showed sRNAs with smaller lengths than predicted for sRNAs ssrS and sra25 (15 nt of difference) as well as for sm84 (30 nt of difference) and sm270 (10 nt of difference) [86]. The sRNA processing requires enzymatic cleavage to remove extra residues by ribonucleases to generate functional stable forms [87]. *H. seropedicae* SmR1 presents RNase E (Hsero_RS09410) an endoribonuclease which preferentially cleaves AU-rich regions [88] and affect sRNA biogenesis [89]. PNPase (Hsero_RS08755) could also trim sRNAs contributing to their maturation and/or degradation [90]. In fact, this difference in size can also account for the differences in expression observed between the RNA-Seq data and the Northern blots.

## Conclusions

We reported the expression of several sRNA in *H. seropedicae* SmR1 genome in the presence of two nitrogen sources and/or in the presence of naringenin. The functions of the novel sRNAs remain unknown but their existence in this bacterium confirms the evidence that sRNAs are involved in many different cellular activities to adapt bacterium to nutritional and environmental changes. Some of them may participate in the regulation of nitrogen metabolism or in the bacterial-plant interaction. The discovery and knowledge of these sRNA molecules in this nitrogen fixation bacterium is very important due to its biotechnological significance.

## Methods

### Bacterial growth

*H. seropedicae* SmR1 (NCBI sequence: NC_014323.1) was grown at 30 °C and with agitation of 120 rpm in NFbHPN medium containing 80 μg/mL streptomycin [91]. Three growth conditions were used: (i) Control condition (CRT), bacteria grown on NFbHPN medium using malate as carbon source and 20 mmol/L NH_4_Cl as nitrogen source; (ii) Naringenin condition (NAR), bacteria grown in NFbHPN medium in the presence of flavonoid naringenin (100 μM); (iii) Nitrate condition (NIT), bacteria grown in NFbHP medium, using malate as carbon source and 10 mmol/L KNO3 as nitrogen source [12, 17].

### Screening of small RNAs by nocoRNAc and Cufflinks

The *H. seropedicae* SmR1 genome was screened with the computational tool nocoRNAc [44] to search sRNAs by features that include promoter sequence, Rho-independent terminator and regions of sequence conservation. Furthermore, regions with high level of transcription, free of encoded proteins, were assessed in three individual RNA-seq data sets using *Cufflinks* [45] to verify the presence/expression of sRNAs. The nocoRNAc (non-coding RNA characterization) tool predicts putative sRNAs based on analyses not limited to intergenic regions. In order to find the location of candidates, firstly the SIDD sites, which are destabilized regions in the genomic DNA, were identified as putative promoter regions. The SIDD calculation was conducted using default values. After that, the putative Rho-independent terminators were predicted by the TranstermHP program, which is integrated in nocoRNAc. The tool was run using the standard protocol, with the option overwrite set up as described in the user guide [44]. The coordinates of SIDD sites with Rho-independent terminator are used for generating a list of putative sRNAs. Thus, the nocoRNAc tools may detect putative sRNAs in whole genome, even those which are encoded antisense from protein genes.

We assessed RNA-seq data to uncover sRNAs based only on read alignments. The reads from RNA-seq were mapped to *H. seropedicae* SmR1 genome with the tRNA, mRNA, rRNA and their 50 flaking nucleotides masked. We used the default parameters on the Cufflinks program to localize transcribed/expressed regions on the genome likely to encode sRNAs.

### Mapping and visualization of sequence reads and analyses of predicted sRNAs

Before mapping the short reads to the *H. seropedicae* SmR1 reference genome, the rRNA sequences were masked using the cross-match program. Recursive trimming of the reads at 5′ and 3′ to 35 nucleotides were performed using a Perl script and the Mate-Paired reads were aligned to the reference genome using the alignment tool Short Read Mapping Program SHRiMP [92]. The program was set up to tolerate 3 mismatches. The maximal number of hits to each read was 1. Samtools [93] was used to convert data into SAM/BAM format. Mapped RNA-seq reads in BAM format were visualized in the genome browse Artemis [94]. A sequence predicted as a sRNA was considered expressed when the minimum read coverage was 5-fold.

### RNA -Seq data analysis

The RNA-seq data sets used in this work are available in the ArrayExpress database (www.ebi.ac.uk/arrayexpress) under accession number E-MTAB-3435 e E-MTAB-3646. Small non-coding RNA expression profiles was obtained with Artemis [95]. We employed an RPKM normalized expression values of three RNA-seq conditions describe in [12, 17] for the heatmap and hierarchical clustering. We used Pearson correlation as distance measure and average linkage as clustering method in Heatmapper [96]. Genome coordinate plot of non-coding RNA was performed with DNAPlotter [97].

### RNA extraction and northern blot analysis

Overnight cultures grown in the CRT, NAR and NIT conditions were diluted in fresh medium to an initial OD_600_ = 0.1 and grown to exponential (OD_600_ 0.7) and stationary phase (10 h of growth). Culture samples were withdrawn and mixed with an equal volume of RNA stop buffer (10 mM Tris at pH 7.2, 5 mM MgCl_2_, 25 mM NaN_3_, and 500 μg/mL chloramphenicol). The total RNA extraction followed the protocol of cell lysis and phenol:chloroform extraction (adapted from [90]. After a precipitation step in ethanol and 300 mM sodium acetate, RNA was resuspended in MilliQ™-water. The integrity of RNA samples was evaluated by agarose gel electrophoresis. When necessary, Turbo DNase (Ambion) treatment following a new phenol: chloroform step was used to remove contaminant DNA. Next, 10–20 μg of total RNA was used to analyse small RNA expression on 10% polyacrylamide gels in TBE 1x buffer. RNA was transferred onto Hybond-N+ membrane (Amersham Biosciences) using TAE 1x as transfer buffer. RNAs were UV cross-linked to the membrane with a UVC 500 apparatus for 3 min (Amersham Biosciences). DNA templates carrying a T7 promoter sequence for in vitro transcription were generated by PCR using genomic DNA of *H. seropedicae* SmR1 and the primers listed in Table 2. Hsnc028 and Hsnc082 were detected by 5′ -end labelling of an antisense primer (Table 2). Radiolabelled probes using rUTP α-^32^P (T7 probes) or ɣ-^32^P ATP (primer probes) were purified on G25 Microspin columns (GE Healthcare). Hybridizations were carried out overnight at 42 °C or 68 °C with the PerfectHyb Plus Hybridization Buffer (Sigma). RNA Decade marker (Ambion) was used when detecting non-coding RNAs up to 150 nt; for longer transcripts, the 100–1000 bp Ladder (Biotools) was used. All radiochemicals were purchased from Perkin-Elmer.

## Supplementary information

**Additional file 1. ***Herbaspirillum seropedicae* SmR1 predicted sRNAs. Expression of the 117 sRNAs of *H. seropedicae* SmR1 are shown in the table by Coverage and RPKM using RNA-seq data under CRT, NAR and NIT conditions. Defined identity sRNAs have the Rfam code listed. All sRNAs have features as the tools by which the sRNA was predicted (nocoRNAc and / or Cufflinks), category (trans-encoded, cis-encoded or riboswitch), percentage of G + C in the sRNA sequence, initial and final position of the sRNA found in the genome of *H. seropedicae* SmR1 as well as the sense of the DNA strand (sense or antisense) and the size of the sRNA.

## Data Availability

The RNA-Seq data analysed during the current study are available in the ArrayExpress database (www.ebi.ac.uk/arrayexpress) under accession number E-MTAB-2842 e E-MTAB-3435.

## Abbreviations

asRNA: Antisense small RNA
caRNA: Cis-encoded; RNA
CRT: Control condition
mRNA: Messenger RNA
NAR: Presence of naringenin condition
NIT: Nitrate condition
RBS: Ribosomal binding site
Rfam: RNA family database
RNA-seq: RNA sequencing
RPKM: Reads per kilobase million
rRNA: Ribosomal RNA
SAH: S-adenosyl- (L) –homocysteine
SAM: S-adenosyl- (L) –methionine
SIDD: Stress-induced (DNA) duplex destabilization
sRNA: Small non-coding RNA
SRP: Signal recognition particle
tmRNA: Transfer messenger RNA
tmRNP: Ribonucleoprotein complex
TPP: Thiamine pyrophosphate
traRNA: Trans-encoded RNA
tRNA: Transfer RNA
tsRNA: Toxic small RNA
